# Contribution of Discretionary Foods and Drinks to Australian Children’s Intake of Energy, Saturated Fat, Added Sugars and Salt

**DOI:** 10.3390/children4120104

**Published:** 2017-12-01

**Authors:** Brittany J. Johnson, Lucinda K. Bell, Dorota Zarnowiecki, Anna M. Rangan, Rebecca K. Golley

**Affiliations:** 1 School of Pharmacy and Medical Sciences, University of South Australia, Adelaide, SA 5000, Australia; brittany.johnson@mymail.unisa.edu.au (B.J.J.); lucy.bell@.unisa.edu.au (L.K.B.); dorota.zarnowiecki@.unisa.edu.au (D.Z.); 2 Centre for Research Excellence in Early Prevention of Obesity in Childhood, Sydney, NSW 2006, Australia; 3 School of Life and Environmental Sciences, Charles Perkins Centre, University of Sydney, Sydney, NSW 2006, Australia; anna.rangan@sydney.edu.au

**Keywords:** dietary intake, energy-dense, nutrient-poor, child obesity prevention

## Abstract

Interventions are required to reduce children’s consumption of discretionary foods and drinks. To intervene we need to identify appropriate discretionary choice targets. This study aimed to determine the main discretionary choice contributors to energy and key nutrient intakes in children aged 2–18 years. Secondary analyses were performed with population weighted, single 24 h dietary recall data from the 2011–2012 National Nutrition and Physical Activity Survey. Cakes, muffins, and slices; sweet biscuits; potato crisps and similar snacks; and, processed meats and sugar-sweetened drinks were relatively commonly consumed and were within the top three to five contributors to per capita energy, saturated fat, sodium, and/or added sugars. Per consumer intake identified cereal-based takeaway foods; cakes, muffins and slices; meat pies and other savoury pastries; and, processed meats as top contributors to energy, saturated fat, and sodium across most age groups. Subgroups of sugar-sweetened drinks and cakes, muffins and slices were consistently key contributors to added sugars intake. This study identified optimal targets for interventions to reduce discretionary choices intake, likely to have the biggest impact on moderating energy intake while also reducing intakes of saturated fat, sodium and/or added sugars.

## 1. Introduction

Discretionary choices are defined as foods and drinks “not needed to meet nutrient requirements and do not fit into the Five Food Groups… when consumed sometimes or in small amounts, these foods and drinks contribute to the overall enjoyment of eating” [1]. Discretionary choices include subgroups of foods and drinks higher in saturated fat, added sugar and/or salt such as cakes, muffins and slices, sweet biscuits (cookies), chocolate, sugar-sweetened drinks, processed meats, potato crisps and similar, hot chips, and takeaway foods (i.e., burgers, pizza, pies). Excess consumption of discretionary choices, by children in Australia [2] and other countries internationally [3,4], can displace intake of food in the Five Food Groups (vegetables, fruit, whole grains, dairy, meats, and alternatives), provide excess energy intake leading to weight gain, and contribute to the development of chronic health conditions [5,6,7].

Discretionary choices are higher in saturated fat, added sugars, and/or salt [1], which can contribute to diet-related chronic diseases such as Type 2 Diabetes and cardiovascular disease [1,8]. They can be energy-dense and contribute to positive energy balance and obesity in children and adolescents [7]. Children who are overweight or obese are at least twice as likely as children of healthy weight to become overweight or obese as adults [9]. The associated negative health effects of overconsumption of energy, saturated fat, added sugars, and salt seen in adults can emerge earlier in life, including insulin resistance, elevated blood cholesterol levels, and elevated blood pressure [8]. Further, eating habits and food preferences are established early in life, and tend to track into adulthood [10]. Establishing healthy eating habits early can reduce the risk of obesity and diet-related chronic disease across the lifespan. Understanding the contribution of discretionary choices to the intake of energy, saturated fat, added sugars, and salt will enable the identification of targets to focus efforts in children and adolescents to improve population nutrient intakes, diet quality, and health.

In the most recent Australian Health Survey, 27% of 5–17 year old children were overweight or obese [11], a rate similar to, or exceeding those found in other countries [12,13,14]. A comparison of intake of discretionary choices by Australian children aged 2–16 years from 1995 to 2007 by Rangan et al. [15] found that despite a slight decrease in energy intake coming from discretionary foods and drinks (40% vs. 35%), intake considerably exceeded recommendations (0–3 serves, or 0–1800 kJ, depending on age, height, and activity level) [7]. Preliminary analyses of the Australian Health Survey data show that 99% of children and adolescents continue to exceed recommendations for discretionary choice intake [11]. No direct comparisons across countries are available given the varying terminology and classifications of discretionary choices, yet studies in the United States and Mexico have reported between 27–30% of children and adolescents total energy intake derived from several subgroups of discretionary choices [3,16].

This study uses data from the 2011–2012 National Nutrition and Physical Activity Survey (NNPAS) to examine the contribution of energy, saturated fat, added sugars and sodium from discretionary foods and drinks to overall energy intake in Australian children and adolescents. This work updates and builds on the findings of Rangan et al. [15], which described the contribution of discretionary choices to energy intake in a similar population, but did not report the contribution of saturated fat, added sugars and sodium from these foods and drinks to the diet. By considering these nutrients (saturated fat, added sugar, and sodium) in addition to energy, this study will be able to identify targets for dietary modification that can have the greatest impact on population intakes, for both obesity and chronic disease prevention. Thus, the aim of this study was to determine the main discretionary choice subgroups that are contributing to energy and key nutrient intakes in children aged 2–18 years, to identify discretionary choice subgroup targets for intervention. A secondary aim was to compare key food/drink contributors to energy and key nutrient targets across age groups in consumers of discretionary choices.

## 2. Materials and Methods

### 2.1. Data Source

This secondary analysis was performed with day one 24 h recall dietary intake data from 2182 children and adolescents aged 2.0–18.9 years in the National Nutrition and Physical Activity Survey (NNPAS) 2011–12 database (4324.0.55.002—Microdata: Australian Health Survey: Nutrition and Physical Activity, 2011–2012, http://abs.gov.au/AUSSTATS/abs@.nsf/Lookup/4324.0.55.002Main+Features12011-12). Ethics approval was not required as all data were provided by the Australian Bureau of Statistics (ABS) in de-identified format. Children were invited to take part in the national survey following recruitment of an adult within the household by stratified multistage area sample of private households [17]. Dietary recalls were performed face-to-face (with older children/adolescents or parent proxy), between May 2011 and June 2012 using the Automated Multiple-Pass Method [18], and food model booklet and standard measures assisted portion size estimates. Detailed procedures can be found in the Australian Health Survey User Guide [17]. The majority of recalls (84%, 2–18 y) were performed via proxy (i.e., by parent/adult) with or without assistance from the child depending on child age. Children aged 14–18 y were the primary respondent, with or without assistance from a parent. In contrast to previous descriptive analysis of Australian children’s discretionary choice intake [15] (2–16 y), we included the addition of the 17–18 year old age group, in line with the Australian Dietary Guidelines for children and adolescents [1].

### 2.2. Classification of Discretionary Choices

Discretionary choices were classified using the discretionary flag list provided by the ABS; the discretionary flag was assigned to minor food groups (five-digit code) and individual food items (eight-digit code) if they met the definition stated in the dietary guidelines as being high in energy, saturated fat, added sugar, or sodium [19]. Subgroups of discretionary choices were formed based on those previously used [15], using the ABS sub-major grouping (three-digit code, e.g., 131 sweet biscuits) or the minor grouping (5-digit code, e.g., 11,501 soft drinks non-cola, 11,503 soft drinks cola, and 11,505 flavoured mineral water formed the sugar-sweetened soft drinks subgroup) when the sub-major grouping was not appropriate. Similar types of food were grouped together as subgroups; for example, processed meats as mixed foods or in mixed dishes including sausages, rissoles or chicken nuggets were grouped as dishes containing processed meats, whereas sliced ham or chicken loaf, were grouped as processed meats. Due to changes in the latest revision of the Australian Dietary Guidelines and the ABS discretionary flag categorization, we included additional subgroups such as ‘processed meats’ and ‘sweet snack bars’. See Appendix A for the list of 19 discretionary subgroups, and respective food codes, which were used in this analysis. 

### 2.3. Dietary Intake Data

Dietary intake data included individual food items, grams consumed, and corresponding nutrient profile based on the Australian Food, Supplement and Nutrient Database, AUSNUT 2013—a specifically designed food composition database [20]. All of the individuals were given population weighting factors based on age, gender, state of residence, and season by the ABS for the all respondent sample [17], and were applied to reported intake data to give a dataset weighted to 4,770,094 children and adolescents (as at 31 October 2011). Primary analyses were performed on dietary intake from all of the reporters, rather than only plausible reporters, due to the inability to factor in child growth and lack of appropriate and standardized cut offs for children without measured physical activity level (PAL) [21,22], and for consistency with the methodology with the ABS.

### 2.4. Sensitivity Analyses

To account for possible extreme under and over reporting, sensitivity analyses (see Supplementary Tables S1 and S2) were performed with only plausible consumers (i.e., sufficient energy intake to sustain life) as per the Goldberg cut off method [23,24]. The Goldberg method compares energy intake (EI) to basal metabolic rate (BMR) ratio with energy expenditure (PAL) [24]. EI:BMR provided in the NNPAS data set was used, with plausible reporters being considered those within two standard deviations of a PAL of 1.55, and cut offs for under reporters as less than 0.87 and over reporters over 2.74. Respondents with missing EI:BMR (*n* = 507, due to missing weight data) were included with plausible reporters, as we were unable to assess if their reported intake was plausible. Three-hundred and fifty children and adolescents were determined to be non-plausible (*n* = 261 under-reporters, *n* = 89 over-reporters), leaving 2462 plausible reporters (*n* = 4,181,497 population weighted).

### 2.5. Statistical Analysis

Analyses were conducted to determine: (1) per capita, and (2) per consumer intake of discretionary choices. Per capita analyses were performed for 2–18 year olds using the summation of grams, energy (kJ, with fibre), saturated fat (g), added sugars (g) and sodium (mg) intake divided by the total population, to give population aggregated mean absolute quantity, energy, and nutrient values. Percentage of total energy and nutrient intake was calculated using the total diet (including all foods and drinks) values for 2–18 year olds of quantity 2402 g, energy 7998 kJ, saturated fat 29 g, added sugar 58 g, and sodium 2313 mg. 

Per consumer absolute intake was calculated using the same approach, but using discrete consumer populations for each discretionary subgroup, based on the number of survey respondents consuming at least one food item within the subgroup. Per consumer data analyses were performed for the whole sample (2–18 year olds) and repeated for the following age groups: 2–3, 4–8, 9–13, 14–16, and 17–18 year olds, with age specific population values. All data presented in the tables are expressed as absolute values. To keep denominators constant quantity, energy, and nutrient percentage of total diet presented for consumers were based on mean total intake for the age group (including consumers and non-consumers).

All of the population sizes, number of consumers and combined quantity, energy, and nutrient values were obtained from the dataset in IBM SPSS Statistics (Version 23; SPSS Inc., Chicago, IL, USA) and were divided by the relevant population, and percentage of total diet calculations were performed in Microsoft Excel 2013 (Microsoft Corporation, Redmond, WA, USA). Results are presented for subgroups that were consumed by more than 5% of the 2–18 year old sample and contributed to 1% or more of total energy, saturated fat, added sugars, or sodium in the overall diet, which is consistent with previous analyses [15].

## 3. Results

### 3.1. Sample Characteristics

Of the 2–18 year olds within the survey sample (*N =* 2182), the average age was 9.5 ± 5.0 years, half (50%) were male, two-thirds (68%) were healthy weight, and approximately one-quarter were overweight (19%) or with obesity (8%), and a quarter were in the highest Socio-Economic Index For Area quintile (25%). Mean population weighted total intake (discretionary and core choices) consisted of 8031 kJ of energy, 29 g of saturated fat, 58 g of added sugars and 2313 mg of sodium.

### 3.2. Per Capita Consumption

Table 1 shows the per capita intake of discretionary choice subgroups by children and adolescents aged 2–18 years. Nearly all (99%) of the children and adolescents consumed at least one discretionary choice subgroup on the day prior to the survey, with sweet biscuits (31%), potato crisps and similar snacks (27%), and sugar-sweetened soft drinks (25%) the being most commonly consumed choices, consumed by over one-quarter of children. Cakes, muffins and slices (4%), sweet biscuits (3%), and potato crisps and similar snacks (3%) were top contributors to energy intake. Cakes, muffins and slices (4%) also contributed largely to saturated fat (5%), and added sugars (11%) intake. The fourth largest contributor to energy intake was dishes containing processed meats (2.5%), which was also a top contributor to saturated fat (5%) and sodium (5%) intake. Other top contributors to energy and nutrient intakes were ice-cream and ice-blocks (5% saturated fat), fruit drinks (8% added sugars), processed meats (6% sodium) and cereal-based takeaway foods (4% sodium).

### 3.3. Per Consumers

#### 3.3.1. Percent of Total Population Consuming

Contribution of discretionary choice subgroups to energy and nutrient intakes of consumers (for 2–18 year olds and by age group) is presented in Table 2. The most commonly consumed discretionary choice by children aged 2–3 and 4–8 years was sweet biscuits (38% and 25%, respectively), when compared to potato crisps and similar snacks (34%) for 9–13 year olds and sugar-sweetened soft drinks for 14–16 year olds and 17–18 years olds (38% and 43%, respectively). 

There were a number of similarities in the percent of the population that were consuming certain subgroups across age groups. Sweet biscuits and processed meats were highly consumed across most age groups, whereas sugar-sweetened soft drinks were commonly consumed in older children (9–18 y). In 2–3 year olds, sweet biscuits (38%), butter and dairy fats (21%), and dishes containing processed meats (19%) were the most commonly consumed. Sweet biscuits (35%), potato crisps and similar snacks (31%), and processed meats (25%) were most commonly consumed by 4–8 year olds. In 9–13 year olds, potato crisps and similar snacks (34%), sweet biscuits (3.1%), and sugar-sweetened soft drinks (29%) were most commonly consumed. Sugar-sweetened soft drinks (37%), processed meats (23%), and sweet biscuits (23%) were most commonly consumed by 14–16 year olds. Similarly, in 17–18 year olds, sugar-sweetened soft drinks (43%), processed meats (25%), and chocolate (21%) were most commonly consumed subgroups.

#### 3.3.2. Contribution to Energy, Saturated Fat, Added Sugars and Sodium Intakes

Contribution to energy intake for all of the 2–18 year olds was greatest for cereal-based takeaway foods (29%), cakes, muffins and slices (24%), meat pies and other savoury pastries (21%), and dishes containing processed meats (16%). These subgroups were also amongst the top contributors to saturated fat (46%, 31%, 33%, 29%, respectively) and sodium (51%, 18%, 33%, 34%, respectively) intake. Sugar-sweetened soft drinks (75%), cordials (68%), and cakes, muffins and slices (61%) were the greatest contributors to added sugars intake.

A similar pattern was observed across age groups. Cereal-based takeaway foods, cakes, muffins and slices, meat pies and other savoury pastries, and processed dishes containing processed meats were the top four contributors to percent energy (all ages), saturated fat (all ages), and sodium (2–3 y, 4–8 y and 9–13 y) intake for most of the age groups. Processed meats were also a major contributor to sodium intake across all of the age groups (21–28%). Consistent key contributors to sugar intake were cordials (49–99%), fruit drinks (24–78%), sugar-sweetened soft drinks (52–72%), and cakes, muffins and slices (38–90%).

**Figure 1:**
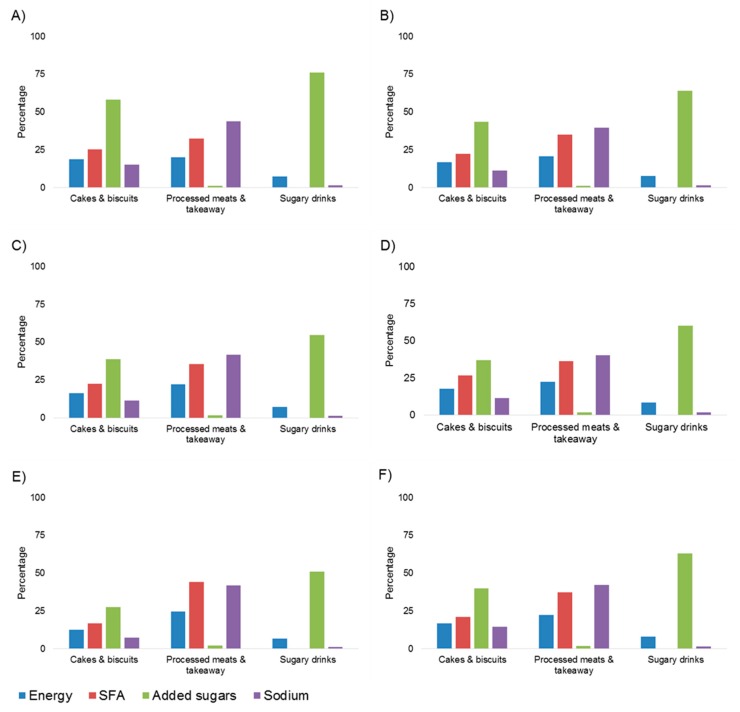
Target discretionary choice subgroups contribution to total percentage energy, SFA, added sugars and sodium, by age group of consumers. Values presented as an average percentage for each condensed subgroup: Cakes & biscuits: cakes, muffins and slices, sweet biscuits; Processed meats & takeaway: dishes containing processed meats, cereal-based takeaway foods; and, Sugary drinks: sugar-sweetened soft drinks, fruit drinks, cordials. (**A**) 2–3 year olds; (**B**) 4–8 year olds; (**C**) 9–13 year olds; (**D**) 14–16 year olds; (**E**) 17–18 year olds; (**F**) 2–18 year olds.

Small differences were also observed across the age groups. The contribution of cereal-based takeaway foods to saturated fat intake increased across age groups, from over one-third (41% and 37%, respectively) in 2–3 year olds and 4–8 year olds, to nearly half (49%) in 17–18 year olds. Similarly, the contribution of sugar-sweetened drinks to added sugars intake was approximately half (52%) in 2–3 year olds when compared to nearly three-quarter (72% and 71%) in 14–16 year olds and 17–18 year olds, respectively. Whilst cakes, biscuits, and muffins were amongst the top four contributors to saturated fat intake for most age groups, chocolate (29%) and ice-cream and ice blocks (27%) were larger contributors for 17–18 year olds. Further, higher fat savoury biscuits (24%) and potato crisps and similar snacks (13%) were greater contributors to sodium intake in 14–16 and 17–18 year olds, respectively, than cakes, muffins and slices (17% and 10%, respectively). Figure 1 shows the relative percentage contribution to total energy, saturated fat, added sugars and sodium, for consumers of condensed common discretionary subgroup targets across the age ranges.

### 3.4. Sensitivity Analyses Excluding Extreme Mis-Reporters

When analyses were undertaken in the sub-sample of plausible reporters, the pattern of results remained consistent (Supplementary Tables S1 and S2). Per capita commonly consumed foods for plausible reporters were sweet biscuits 32%, potato crisps and similar snacks 28%, and sugar-sweetened soft drinks 24%. In the plausible reporter analyses, processed meats emerging as the third most common subgroup (25%) and also the top contributor to energy intake (4%). Cakes, muffins and slices remained a large contributor to energy (4%), saturated fat (5%), and added sugars (9%) intake. Top subgroups contributing to saturated fat, added sugar and sodium were comparable to all of the reporters with the exception of sweet snack bars identified as the largest contributor to saturated fat (5%).

Per consumer plausible reporters by age group again had similar patterns to all reporters. Commonly consumed foods primarily remained within the top three subgroups across age groups, however within adolescents (14–18 y) dishes containing processed meats emerged as the second most commonly consumed (31–41%). Cereal-based takeaway foods (26%), cakes, muffins and slices (20%), meat pies and other savoury pastries (19%), and dishes containing processed meats (14%) remained the largest contributors to total energy intake in 2–18 year olds, and across age groups. These subgroups were still amongst the top contributors to saturated fat and sodium (except for cakes, muffins and slices), across age groups. Sugar-sweetened soft drinks (52–61% 4–18 y) or fruit drinks (72% 2–3 y), cordials (39–82%) and cakes, muffins and slices (30–63%) were consistently high contributors to added sugar, across age groups and all reporters.

## 4. Discussion

This analysis of a nationally representative sample of Australian 2–18 year olds describes the specific discretionary food and beverage groups that contribute most to total energy, saturated fat, sodium, and added sugars intakes. Cakes and biscuits, cereal-based takeaway foods, dishes containing processed meats, and sugary drinks were relatively commonly consumed and were within the top three to five sources of energy, saturated fat, sodium, and/or added sugars. While some minor differences were observed by age group sub-categories, cakes and biscuits were ubiquitous in their contribution to dietary intake across age group categories. Reducing the intake of selected discretionary choices, such as cakes and biscuits, is likely to provide the biggest impact across multiple dietary components.

Discretionary choices are a heterogenous collection of foods and drinks that are not needed to meet nutrient requirements, and are associated with higher intakes of energy and nutrients that increase risk of obesity and multiple chronic diseases [1]. Children’s overall discretionary choices intake may be high in energy, saturated fat, sodium, or added sugars, but subgroups of discretionary choices are not necessarily high across each of these dietary targets. The novelty of the present analysis is in consideration of multi-nutrient analysis; by identifying top contributors across the dietary factors of concern, selected discretionary choice subgroups may be prioritized for nutrition messages. Focusing interventions on a targeted group of discretionary choices may deliver the best impact on population nutrient intakes, diet quality and health.

Cakes and biscuits, and processed meats (and to a lesser extent cereal-based takeaway foods) were commonly consumed and were top contributors to dietary factors of concern. Collectively, all sugar-sweetened drinks (i.e., cordials, fruit juice drinks and soft drinks, energy and sports drinks) were also commonly consumed, were the top contributor to added sugars, and contributed a combined 4.4% to total energy intake. In contrast, cakes, muffins, and slices alone contributed 4.2% to total energy intake. Similar patterns were generally observed across the per capita, per consumer, and plausible reporter analyses. Targeting reductions in intakes of these five (of 19) (cakes, muffins and slices; sweet biscuits; processed meats; cereal-based takeaway foods; and, sugar-sweetened drinks (including fruit drinks and cordials) groups of discretionary foods and drinks would appear to have the biggest impact across total energy intake, as well as saturated fat, sodium, and added sugars intakes. Targeting consumers of these discretionary choices is likely to achieve a significant overall population impact.

The analysis by age group categories revealed small differences in patterns between younger and older children; however the same food groups remained broadly the top contributors to energy and nutrient intakes. While intake trends increased across the age groups, this is likely to reflect increased requirements with growth. Focusing on the percent of total intake, the trends were similar across age groups, perhaps except for sugar-sweetened soft drinks and cereal-based takeaway foods. Cakes and biscuits, processed meats, butter and dairy fats, and fruit drinks were commonly consumed in the 2–3 year old group. Consumption patterns for commonly consumed discretionary choices are evident from a young age and are relatively stable across the age group categories. The higher total discretionary choice intake in older children and adolescents reflects stable trends in key discretionary choices (such as cakes and biscuits), plus the introduction of additional subgroups (e.g., soft drinks, chocolate and fried potatoes) during adolescence.

This study utilizes similar methodology to Rangan et al. [15], which describes intake of ‘extra’ foods in Australian 2–16 year olds in 1995 and 2007. There are differences with the present analyses in terms of the definition of ‘extra’ versus ‘discretionary’ choices (reflecting changes in dietary guidelines between 2003 [25] and 2013 [1]), the age range of the sample analyzed, and other variances in survey methodology preventing direct comparison to Rangan et al. [19]. However, the percent of total energy intake from cakes, muffins, and slices is higher in 2011–2012 (i.e., this study) compared to 2007 sample (4.2% vs. 2.9%) [15]. Percent of energy derived from fried potato is slightly lower (2.5% in 2011–2012 vs. 2.9% in 2007), whereas the contribution to total energy intake of sweet biscuits has slightly increased (2.9% vs. 2.5%), and potato crisps (2.7% vs. 2.6%) have remained the same [15]. Processed meats were not classified as ‘extra’ foods in the previous analysis. In the present survey, these subgroups contributed 5.2% and 10.7% of total energy and sodium intake, respectively. Importantly, in the present analysis, contribution to saturated fat, sodium and added sugars intake are described enabling interpretation beyond gram and energy intake. Children’s intake of discretionary choices remains well above recommended intakes, with little qualitative change being observed since 2007. Interventions are urgently needed that achieve reductions in the intake of discretionary choices. The benefit of targeting the discretionary choices highlighted in this analyses, is that there is likely to be benefit in terms of reduced risk of positive energy balance and risk of obesity, together with risk factor reduction supporting the prevention of conditions, such as diabetes, dental caries, hypertension, and cardiovascular disease.

There remain few international studies that examine contemporary consumption patterns of discretionary-type foods and drinks, particularly in the context of considering energy intake and multiple nutrients of concern [26,27,28]. In a United States (U.S.) study using data from the 2003–2006 National Health and Nutrition Examination Survey (NHANES), cake/cookies/pastry-type foods were a top ranked source of total energy intake and soft drinks/soda and candy/sugar/sugary foods were the top two sources of added sugars in 2–18 year olds [3]. Another U.S. study, using this same data, identified overlap between major sources of energy and ‘empty calories’ including soda, grain desserts and pizza [29]. Cakes, cookies, desserts, as well as sugary drinks and savory snacks, were major sources of energy intake in a nationwide survey of Brazilians (10 years and older) [26]. The findings from the present study show cakes and biscuits and sugary drinks as top contributors to total energy and added sugars intake, but also highlight that cakes and biscuits and additional groups, such as processed meats and cereal-based takeaway foods, further make a significant contribution to energy, saturated fat, sodium, and added sugars intake.

With an understanding of key discretionary food and beverage targets, the dietary strategies that can be leveraged to reduce children’s discretionary choice intake can be considered. For example, Van de Bend et al. [30] describe the changes in portion sizes between the 2007 and 2011–2012 Australian Health Surveys. Between these years, the portion size of processed meats, such as sausages, increased, while the portion sizes of many energy-dense packaged snack foods has remained stable (e.g., muesli bars, potato crisps, chocolate) or increased (e.g., meat-based dishes and cakes) [30]. Interventions to reduce discretionary choice portion size, such as downsizing serving sizes of packaged foods, are important but will require further focus beyond efforts to date [31]. Dietary strategies, such as reducing the frequency of consumption of discretionary choices or via substitution to choices within the five food groups, are warranted [32]. Recent modelling in adults has shown substitution to be useful [33], perhaps signaling a revisit of a swap-it type campaign drawing on the stronger evidence base now available [34]. The multi-strategy approach of the Australian Healthy Food Partnership [35] is important, with the moderation of portion size, substitution for healthy foods, and to a lesser degree reformulation of discretionary choices are all important strategies to leverage. Reformulation may have a role to play in reducing added sugars, sodium, and saturated fat intakes, but has limits in its food science technology to manipulate energy density [33]. Interventions spanning the socio-ecological model, for example, the food supply, food availability, and importantly, social norms, will be needed to address these key targets [36].

This secondary analysis of Australia’s most contemporary national nutrition survey data has some strengths and limitations. The strengths include analysis applying population weighted food intake to account for location, age, and gender. The survey under represents Friday and Saturday intakes, which may mean that discretionary choices intake is underreported across the population. This was the same in previous surveys. Categorization was based on the ABS discretionary choices flag, which while having some anomalies, enables comparison with ABS data and other studies using the same flag. The analysis utilized the definition of discretionary choices that were reflected in the current 2013 Australian Dietary Guidelines [1]. Limitations include those that were related to the methodology of the National Nutrition and Physical Activity Survey dataset, including the use of 24 h dietary recalls [17]. Misreporting including both under- and over-reporters was considered in conducting a sensitivity analysis excluding implausible reporters identified based on the Goldberg method [23,24]. This has been presented as a supplementary analysis given misreporting within plausible energy intakes is likely and current methods do not overcome this. It is unclear if excluding implausible reporters addresses measurement bias or introduces alternative bias [22]. Only the first 24 h recall data was used, and therefore the results reflect group patterns not necessarily usual intake. This is not an issue in our analysis as no comparisons with reference values were undertaken. While longitudinal analysis to formally evaluate changes in consumption patterns as compared to 1995 and 2007 survey data would be ideal, lack of resources and issues with differences in survey methodology (question validity) prevent this at the current time.

## 5. Conclusions

In conclusion, Australian children and adolescents aged 2–18 years continue to source nearly 40% of their daily energy intake from discretionary foods that are high in saturated fat, sodium, and/or added sugars. Cakes and biscuits, cereal-based takeaway foods, dishes containing processed meats, and sugary drinks are the optimal targets for interventions to reduce discretionary choices intake. These food targets are likely to have the biggest impact to moderate energy intake, while also reducing intakes of saturated fat, sodium, and/or added sugars.

## Figures and Tables

**Table 1:** Mean per capita intake of discretionary choice subgroups by children and adolescents aged 2–18 y in NNPAS 2011–12 (*n* = 4,770,094 ^1^).

Food Groupings	% Consuming	Weight (g)	% of Total Weight ^2^	kJ	% of Total Energy ^3^	SFA (g)	% of Total SFA ^3^	Added Sugar (g)	% of Total Added Sugar ^3^	Sodium (mg)	% of Total Sodium ^3^
Cakes, muffins and slices	17.4	22.3	0.9	335.7	4.2	1.6	5.4	6.2	10.6	71	3.1
Sweet biscuits	30.6	11.1	0.5	227.9	2.9	1.0	3.4	3.3	5.7	83	3.6
Potato crisps and similar snacks	26.6	28.3	1.2	211.6	2.7	1.0	3.4	0.1	0.2	62	2.7
Dishes containing processed meats	15.5	18.4	0.8	198.1	2.5	1.3	4.5	0.0	0.0	120	5.2
Fried potatoes	18.4	18.7	0.8	197.2	2.5	0.6	1.9	0.0	0.0	35	1.5
Ice cream and ice blocks	20.2	22.3	0.9	179.1	2.2	1.5	5.2	3.3	5.6	12	0.5
Sugar-sweetened soft drinks	24.8	112.5	4.7	173.9	2.2	0.0	0.0	10.8	18.6	16	0.7
Meat pies and other savoury pastries	10.5	18.0	0.8	172.9	2.2	1.0	3.5	0.2	0.4	79	3.4
Cereal-based takeaway foods	7.2	15.3	0.6	166.0	2.1	1.0	3.3	0.2	0.3	85	3.7
Chocolate	18.7	7.8	0.3	162.8	2.0	1.3	4.4	3.4	5.8	7	0.3
Higher-fat savoury biscuits	11.2	4.9	0.2	103.2	1.3	0.5	1.8	0.2	0.4	40	1.7
Fruit drinks	17.4	55.5	2.3	96.3	1.2	0.0	0.0	4.7	8.0	3	0.2
Sweet snack bars	16.5	5.5	0.2	92.5	1.2	0.3	1.0	0.9	1.6	7	0.3
Processed meats	23.9	9.8	0.4	84.8	1.1	0.5	1.6	0.1	0.1	128	5.5
Cordials	9.9	43.2	1.8	65.8	0.8	0.0	0.0	3.9	6.7	3	0.1
Butter and dairy fats	21.4	1.9	0.1	54.8	0.7	0.9	3.1	0.0	0.0	12	0.5
Lollies and confectionary	15.7	3.7	0.2	53.3	0.7	0.0	0.1	2.2	3.7	4	0.2
All sugar-sweetened drinks	45.8	221.9	9.2	349.0	4.4	0.0	0.0	20.1	34.7	26	1.1
All discretionary choices	98.6	477.6	19.9	3079.7	38.5	14.2	49.0	50.3	86.7	938	50.6

NNPAS: National Nutrition and Physical Activity Survey; SFA: saturated fatty acids. ^1^ Percent consuming of survey respondents (*N* = 2812), mean intakes population weighted. ^2^ Total food and beverage weight (i.e., contribution to total intake) is 2402.2 g. ^3^ Total energy and nutrients intake (i.e., contribution to total core and discretionary food intakes) are 7997.9 kJ, SFA 29.1 g, added sugars 58.0 g, sodium 2312.7 mg.

**Table 2:** Mean quantities consumed per consumers of discretionary choice subgroups by age group in the NNPAS 2011–2012 survey ^1^.

**Food Subgroup**	**% of Total Population Consuming ^2^**						**Quantity (g) per Consumer**						**Energy (kJ)**					
	2–3 y	4–8 y	9–13 y	14–16 y	17–18 y	All 2–18 y	2–3 y	4–8 y	9–13 y	14–16 y	17–18 y	All 2–18 y	2–3 y	4–8 y	9–13 y	14–16 y	17–18 y	All 2–18 y
Cakes, muffins and slices	13.6	19.1	19.6	17.3	12.1	17.4	108.1	118.9	135.1	147.9	98.2	128.7	1638.8	1755.0	2022.0	2306.1	1441.2	1934.2
Sweet biscuits	37.5	35.0	31.4	23.0	17.8	30.6	32.8	31.9	40.9	49.1	42.0	36.3	618.6	619.4	808.2	981.3	822.0	744.3
Potato crisps and similar snacks	17.0	31.3	33.5	21.7	18.6	26.6	32.2	39.5	37.8	50.5	51.4	41.7	645.3	795.8	784.6	1046.3	1058.1	855.6
Dishes containing processed meats	19.0	18.0	15.1	10.8	11.7	15.5	72.3	117.4	124.8	140.3	168.9	118.9	785.5	1242.8	1350.0	1526.5	1855.4	1280.5
Fried potatoes	16.2	17.4	18.6	20.9	20.1	18.4	61.0	60.1	104.3	164.2	119.5	101.7	640.8	661.8	1106.6	1683.6	1260.7	1072.5
Ice cream and ice blocks	14.0	22.8	24.9	17.9	13.6	20.2	54.4	85.1	119.8	136.8	148.9	110.5	411.8	694.0	950.8	1124.8	1168.9	886.4
Sugar-sweetened soft drinks	6.0	16.9	29.4	37.4	43.2	24.8	188.9	283.9	418.3	550.4	512.1	454.7	269.7	419.1	646.1	863.6	797.3	702.5
Meat pies and other savoury pastries	7.1	9.1	11.4	13.6	11.4	10.5	132.3	171.6	153.9	209.7	156.6	171.7	1196.0	1541.9	1535.2	2079.6	1525.8	1653.3
Cereal-based takeaway foods	5.0	4.9	7.0	10.4	11.7	7.1	147.0	160.9	221.7	238.3	239.5	213.7	1628.7	1675.8	2454.9	2580.1	2603.9	2322.3
Chocolate	15.3	18.1	19.8	19.7	21.2	18.7	32.8	30.8	46.8	43.1	53.9	41.7	686.8	635.0	971.4	912.4	1134.2	870.3
Higher-fat savoury biscuits	11.4	12.8	11.3	10.8	6.4	11.2	22.4	32.1	46.9	80.9	38.1	44.0	462.3	671.9	980.1	1703.4	796.7	921.1
Fruit drinks	17.0	19.9	17.7	14.6	14.8	17.4	295.9	292.0	337.6	384.4	277.0	320.0	521.7	501.1	581.6	679.8	484.3	555.1
Sweet snack bars	11.0	18.3	19.8	17.9	8.3	16.5	34.9	32.0	30.7	38.9	27.7	33.3	581.9	533.2	514.4	658.0	488.4	560.3
Processed meats	18.8	24.8	26.0	23.2	25.0	23.9	21.9	36.4	41.0	51.2	49.4	41.1	174.1	298.9	346.6	488.2	435.1	354.7
Cordials	10.3	9.8	11.3	8.9	6.8	9.9	362.3	488.3	409.9	504.2	366.6	438.2	542.3	718.7	627.7	809.5	579.1	668.2
Butter and dairy fats	21.3	22.1	23.3	18.9	18.9	21.4	7.5	7.0	8.9	13.7	6.0	8.7	222.8	205.2	262.8	406.0	179.2	255.8
Lollies and confectionary	14.7	17.5	17.3	14.4	9.8	15.7	16.3	20.7	25.5	31.0	20.3	23.6	230.5	290.2	378.4	443.5	283.8	339.7
All sugar-sweetened drinks	29.5	41.0	50.8	55.3	56.4	45.8	336.2	380.4	464.7	601.7	567.7	484.5	546.0	594.3	730.8	952.2	881.6	762.0
All discretionary choices	96.8	98.9	99.0	98.8	99.6	98.6	248.3	367.6	511.1	658.0	693.7	484.3	1858.1	2677.3	3422.3	4015.7	3338.1	3123.1
**Food Subgroup**	**Saturated Fatty Acids (g)**						**Added Sugars (g)**						**Sodium (mg)**					
	2–3 y	4–8 y	9–13 y	14–16 y	17–18 y	All 2–18 y	2-3 y	4-8 y	9–13 y	14–16 y	17–18 y	All 2–18 y	2–3 y	4–8 y	9–13 y	14–16 y	17–18 y	All 2–18 y
Cakes, muffins, slices	8.1	7.9	9.4	11.8	5.9	9.1	29.1	33.0	37.7	40.1	26.4	35.5	354	375	440	459	289	409
Sweet biscuits	3.1	3.4	4.7	6.0	4.8	3.2	8.6	9.0	12.1	14.4	12.3	10.7	95	95	128	152	123	270
Potato crisps and similar snacks	3.0	3.8	3.3	4.3	4.3	3.7	0.2	0.4	0.2	0.6	1.1	0.4	237	315	271	363	362	310
Dishes containing processed meats	5.2	8.4	8.9	9.4	12.7	8.5	0.0	0.1	0.0	0.0	0.1	0.1	480	790	832	810	1057	774
Fried potatoes	1.9	2.0	2.9	4.3	4.2	3.0	0.0	0.0	0.0	0.0	0.0	0.0	86	110	184	326	245	189
Ice cream and ice blocks	3.3	6.1	8.0	9.3	8.7	7.4	7.9	12.3	17.4	20.5	21.7	16.1	27	46	64	76	78	60
Sugar-sweetened soft drinks	0.0	0.0	0.0	0.0	0.0	0.0	16.7	26.0	40.1	53.7	49.7	43.7	30	44	60	75	64	63
Meat pies and other savoury pastries	6.8	8.4	9.5	11.9	9.0	9.6	2.5	1.5	2.3	2.8	2.5	2.3	539	695	715	890	779	753
Cereal-based takeaway foods	9.1	9.4	13.6	15.0	15.6	13.3	0.9	1.2	2.0	2.7	2.8	2.1	819	837	1220	1376	1313	1182
Chocolate	5.5	5.0	7.5	7.3	9.3	6.9	13.8	13.7	20.1	18.3	23.2	18.0	26	29	49	35	51	39
Higher-fat savoury biscuits	2.3	3.4	5.0	8.9	4.0	4.7	0.8	1.5	2.1	4.1	2.0	2.1	178	256	381	663	290	355
Fruit drinks	0.0	0.0	0.0	0.0	0.0	0.0	25.3	24.3	28.1	32.3	23.7	26.8	16	19	21	22	21	20
Sweet snack bars	1.9	1.7	1.4	1.9	1.3	1.7	6.0	5.7	5.3	6.3	4.7	5.7	44	43	40	45	41	43
Processed meats	0.9	1.7	1.9	2.7	2.3	2.0	0.1	0.2	0.3	0.4	0.3	0.3	275	439	508	763	690	536
Cordials	0.0	0.0	0.0	0.0	0.0	0.0	31.9	42.2	36.9	47.5	34.1	39.3	27	33	27	36	22	30
Butter and dairy fats	3.6	3.4	4.3	7.0	3.1	4.3	0.0	0.0	0.0	0.0	0.0	0.0	44	47	58	101	43	58
Lollies and confectionary	0.1	0.1	0.4	0.2	0.0	0.2	9.6	11.6	15.1	18.7	11.1	13.7	20	24	29	41	24	28
All sugar-sweetened drinks	0.0	0.0	0.0	0.0	0.0	0.0	29.2	33.0	42.1	56.0	52.9	43.9	25	36	53	81	84	57
All discretionary choices	8.7	12.7	16.2	18.3	14.0	14.4	27.8	41.3	56.3	66.7	60.8	51.0	542	854	1003	1226	1054	952

^1^ Mean gram, energy and nutrient intake of population weighted consumer population. ^2^ Percent consuming of survey respondents.

## Supplementary Materials

The following are available online at www.mdpi.com/2227-9067/4/12/104/s1, Table S1: Mean per capita intake of discretionary choice subgroups by plausible reporters aged 2–18 y in NNPAS 2011–12 (n = 4,181,497), Table S2: Mean quantities consumed per-consumers of discretionary choice subgroups by age group in the NNPAS 2011–2012 survey of plausible reporters.

## Appendix A

**Table A1:** Discretionary subgroup classifications using the Australian Bureau of Statistics Food Codes.

Subgroup Name	Food Codes ^1^ and Examples
Cakes, muffins, slices	133 Cakes, muffins, scones, cake-type desserts
Sweet biscuits	131 Sweet biscuits
Potato crisps and similar snacks	261 Potato snacks, 262 Corn snacks, 263 Extruded or reformed snacks, 264 Other snacks
Dishes containing processed meats	155 Fish and seafood products (crumbed/battered fish), 185 Sausages, 187 Mixed dishes where beef is main ingredient (patty), 188 Mixed dishes where sausage/bacon is main ingredient (dippy dog), 189 Mixed dishes where chicken is main ingredient (chicken nuggets, wings)
Fried potatoes	241 Potatoes, 24102 Potato products
Ice cream and ice blocks	195 Frozen milk products (ice cream, frozen yoghurts, frozen dairy desserts), 204 Soy-based ice confection, 27303 water ice confection, gelato, sorbet
Sugar-sweetened soft drinks	11501, 11503, 11505 Soft drinks and flavoured mineral waters (excluding intense sweetened)
Meat pies and other savoury pastries	13404, 13405, 13406 Savoury pastry products (quiches, pies, rolls)
Cereal-based takeaway	135 Mixed dishes where cereal is the main ingredient (e.g., pizza, burger, spring rolls, fried rice, pasta)
Chocolate	281 Chocolate and chocolate-based confectionary
Higher-fat savoury biscuits	132 Savoury biscuits
Fruit drinks	113 Fruit drinks (ready to drink or made from concentrate), vegetable drinks, fruit drinks prepared from dry powder
Sweet snack bars	283 Muesli or cereal style bars, 282 Fruit, nut, seed bars
Processed meats	186 Processed meats
Cordials	11401, 11403 Cordial concentrate and made (excluding intense sweetened)
Butter and dairy fats	141 Butters, 142 Dairy blends
Lollies and confectionary	284 Other confectionary
All sugar-sweetened drinks	11401, 11403 Cordial, 11501, 11503, 11505 Soft drinks and flavoured mineral waters, 113 Fruit drinks, 11601, 11602 Sports drinks, 11603 Energy drinks
All discretionary choices	All discretionary flagged foods and drinks

**^1^** Only individual food items (8-digit code) flagged as discretionary choices were captured. Food codes presented at the sub-major (3-digit code) or minor (5 digit code).
