# Supplementary material for: Contribution of Discretionary Foods and Drinks to Australian Children’s Intake of Energy, Saturated Fat, Added Sugars and Salt
Source: Children (Basel). 2017 Dec 1;4(12):104. doi: 10.3390/children4120104 (PMC5742749; doi:10.3390/children4120104)
Supplement: Supplementary file 1 [file children-04-00104-s001.pdf]

Supplementary Table S1: Mean per capita intake of discretionary choice subgroups by plausible [1] reporters aged 2–18 y in NNPAS 2011-12 (n = 4,181,497).

Food Subgroup	% Consuming	Weight (g)	% of Total Weight [2]	kJ	% of Total Energy [3]	SFA (g)	% of Total SFA	Added Sugar (g)	% of Total Added Sugar	Sodium (mg)	% of Total Sodium
Cakes, muffins and slices	17.8	22.1	0.8	331.0	3.6	1.5	4.7	6.1	9.2	70	2.7
Sweet biscuits	31.7	11.7	0.4	229.6	2.5	1.3	3.9	3.4	5.1	36	1.4
Potato crisps and similar snacks	27.9	11.6	0.4	236.8	2.6	1.0	3.1	0.1	0.2	86	3.3
Processed dinner meats	15.6	18.0	0.7	193.1	2.1	1.3	3.9	0.0	0.0	117	4.5
Fried potatoes	18.4	18.4	0.7	194.0	2.1	0.5	1.6	0.0	0.0	35	1.3
Ice cream and ice blocks	20.9	23.2	0.9	186.2	2.0	1.6	4.7	3.4	5.1	12	0.5
Sugar-sweetened soft drinks	24.3	113.2	4.1	175.0	1.9	0.0	0.0	10.9	16.4	16	0.6
Meat pies and other savoury pastries	10.4	18.7	0.7	179.2	2.0	1.0	3.2	0.3	0.4	81	3.1
Cereal-based takeaway	7.1	15.6	0.6	169.4	1.9	1.0	2.9	0.2	0.2	85	3.2
Chocolate	19.2	8.0	0.3	167.9	1.8	1.3	4.0	3.5	5.2	8	0.3
Higher-fat savoury biscuits	11.7	5.3	0.2	110.7	1.2	0.6	1.7	0.3	0.4	43	1.6
Fruit drinks	17.8	57.3	2.1	99.1	1.1	0.0	0.0	4.8	7.2	4	0.1
Sweet snack bars	17.2	5.7	0.2	94.6	1.0	1.6	5.0	1.0	1.5	42	1.6
Luncheon meats	24.6	40.8	1.5	354.5	3.9	0.5	1.5	0.1	0.1	131	5.0
Cordials	10.0	43.3	1.6	65.9	0.7	0.0	0.0	3.9	5.9	3	0.1
Butter and dairy fats	22.3	1.9	0.1	57.3	0.6	1.0	2.9	0.0	0.0	13	0.5
Lollies and confectionary	16.0	3.8	0.1	54.8	0.6	0.0	0.1	2.2	3.4	5	0.2
All sugar-sweetened drinks	45.7	222.5	8.1	350.5	3.8	0.0	0.0	20.2	30.5	25	1.0
All discretionary choices	99.0	480.8	17.5	3125.3	34.3	14.5	43.8	51.0	77.0	950	36.0

Abbreviations: NNPAS: National Nutrition and Physical Activity Survey; SFA: saturated fatty acids.

[1] Percent consuming of plausible survey respondents (n = 2462), mean intakes population weighted (n = 4,181,497 population weighted from plausible respondents).

[2] Total food and beverage weight of plausible reporters (i.e., contribution to total intake) – base case total 2740.4 g.

[3] Contribution to total (core and discretionary foods) plausible reporters based on 9123.7 kJ, SFA 33.1 g, added sugars 66.2 g, sodium 2638.3 mg.

Supplementary Table S2: Mean quantities consumed per-consumers of discretionary choice subgroups by age group in the NNPAS 2011-12 survey of plausible reporters [1].

Food Subgroup	% of Total Population Consuming [2]						Quantity (g)						Energy (kJ)					
	2-3 y	4-8 y	9-13 y	14-16 y	17-18 y	All 2-18 y	2-3 y	4-8 y	9-13 y	14-16 y	17-18 y	All 2-18 y	2-3 y	4-8 y	9-13 y	14-16 y	17-18 y	All 2-18 y
Cakes, muffins and slices	12.8	18.7	19.7	20.0	14.1	17.8	86.0	111.8	133.2	144.5	97.3	123.9	1295.2	1648.3	1986.8	2230.9	1448.1	1856.5
Sweet biscuits	38.3	35.2	32.3	24.1	18.9	31.7	28.5	31.2	41.6	49.0	47.1	36.9	540.4	604.1	823.4	981.1	923.3	723.8
Potato crisps and similar snacks	17.1	31.4	34.6	24.4	20.9	27.9	29.1	39.6	38.4	49.1	49.4	41.5	576.2	795.6	797.6	1017.4	1014.4	849.8
Processed dinner meats	26.4	6.7	17.1	31.3	40.7	15.6	66.6	119.6	116.7	128.6	170.5	115.0	727.3	1262.0	1256.2	1390.7	1876.9	1234.8
Fried potatoes	15.2	16.5	19.6	21.2	21.8	18.4	49.0	56.4	105.8	170.6	107.6	100.2	524.8	603.4	1120.6	1731.6	1181.6	1054.2
Ice cream and ice blocks	13.5	23.4	25.6	19.5	14.1	20.9	53.8	83.9	120.8	133.6	163.5	110.7	407.6	682.5	968.9	1090.3	1274.0	889.9
Sugar-sweetened soft drinks	6.7	16.3	29.6	36.6	46.1	24.3	194.1	290.7	422.9	556.6	555.4	465.1	277.2	431.1	653.2	876.0	864.5	719.1
Meat pies and other savoury pastries	6.7	9.3	11.4	13.7	11.7	10.4	141.4	173.2	157.9	227.4	163.0	179.4	1269.3	1546.0	1582.4	2262.4	1558.8	1722.9
Cereal-based takeaway	4.6	4.6	7.4	10.5	13.1	7.1	157.5	170.1	231.2	215.4	257.6	219.6	1744.7	1757.9	2562.8	2319.8	2802.1	2382.6
Chocolate	15.7	18.0	20.2	21.0	23.8	19.2	27.0	29.6	49.0	43.1	57.4	41.9	566.7	611.1	1012.6	920.1	1210.2	874.1
Higher-fat savoury biscuits	11.6	13.3	11.7	11.7	5.8	11.7	24.1	32.2	49.6	80.4	47.4	45.3	497.4	674.5	1039.1	1693.6	989.9	949.6
Fruit drinks	16.9	19.9	18.3	15.1	16.0	17.8	311.9	301.4	324.5	402.1	251.0	321.1	552.2	516.8	556.9	709.7	437.0	556.0
Sweet snack bars	11.3	18.3	20.6	19.8	8.3	17.2	31.2	31.8	31.0	37.9	26.4	32.9	517.8	530.2	518.1	634.6	451.7	550.7
Luncheon meats	19.0	25.1	26.8	25.9	24.3	24.6	21.7	36.4	41.1	50.9	48.3	40.8	176.3	297.3	343.1	494.7	452.1	354.5
Cordials	10.1	9.7	11.0	9.5	7.8	10.0	367.4	482.0	413.5	471.5	376.1	435.1	517.7	711.5	634.0	770.0	596.3	662.1
Butter and dairy fats	21.9	22.7	24.2	19.3	20.9	22.3	7.5	7.0	8.6	15.2	6.3	8.7	221.4	205.8	253.2	451.1	187.3	257.6
Lollies and confectionary	15.4	17.3	17.5	14.6	10.2	16.0	16.9	19.7	25.5	34.5	20.8	23.7	238.6	277.2	380.2	495.1	288.2	342.5
All sugar-sweetened drinks	29.6	40.3	51.2	55.1	59.2	45.7	347.2	385.6	463.1	598.8	599.6	487.3	554.1	604.3	728.1	956.2	932.1	767.8
All discretionary choices	97.6	99.0	99.4	99.3	100.0	99.0	241.1	363.8	518.2	666.5	751.3	485.5	1708.9	2613.3	3509.6	4207.0	3642.4	3156.0

NNPAS: National Nutrition and Physical Activity Survey.

[1] Mean gram, energy and nutrient intake of population weighted plausible consumer population.

[2] Percent consuming of plausible survey respondents

Supplementary Table S2 (continued): Mean quantities consumed per-consumers of discretionary choice subgroups by age group in the NNPAS 2011-12 survey of plausible reporters [1].

Food Subgroup	Saturated Fatty Acids (g)						Added Sugars (g)						Sodium (mg)					
	2-3 y	4-8 y	9-13 y	14-16 y	17-18 y	All 2-18 y	2-3 y	4-8 y	9-13 y	14-16 y	17-18 y	All 2-18 y	2-3 y	4-8 y	9-13 y	14-16 y	17-18 y	All 2-18 y
Cakes, muffins and slices	6.2	7.5	9.2	11.1	6.0	8.7	23.5	31.2	36.7	38.4	26.5	34.0	281	349	437	452	286	395
Sweet biscuits	2.8	3.3	4.8	5.9	5.5	4.1	7.5	8.7	12.3	14.4	13.7	10.6	83	94	131	154	139	113
Potato crisps and similar snacks	2.6	3.8	3.3	4.2	4.1	3.7	0.2	0.4	0.2	0.6	1.2	0.4	222	317	273	351	340	308
Processed dinner meats	4.9	8.3	8.5	8.6	13.1	8.2	0.0	0.2	0.0	0.0	0.1	0.1	446	799	790	719	1081	750
Fried potatoes	1.6	1.7	3.0	4.2	4.0	2.9	0.0	0.0	0.0	0.0	0.0	0.0	74	101	188	339	229	188
Ice cream and ice blocks	3.3	6.0	8.3	8.9	9.4	7.5	8.0	12.1	17.7	20.1	23.8	16.3	26	46	66	73	84	60
Sugar-sweetened soft drinks	0.0	0.0	0.0	0.0	0.0	0.0	17.2	26.7	40.6	54.4	53.9	44.7	30	45	60	74	69	64
Meat pies and other savoury pastries	7.1	8.4	9.8	12.9	9.3	10.0	2.9	1.4	2.5	3.1	2.5	2.4	561	690	736	945	788	775
Cereal-based takeaway	9.7	9.9	14.2	13.7	16.8	13.6	0.9	1.3	2.1	2.6	2.8	2.1	879	875	1269	1199	1416	1199
Chocolate	4.6	4.8	7.8	7.4	9.9	6.9	11.6	13.1	20.9	18.3	24.8	18.0	20	28	52	32	53	39
Higher-fat savoury biscuits	2.5	3.5	5.3	8.8	5.0	4.9	0.9	1.5	2.2	4.1	2.5	2.1	192	258	406	660	359	367
Fruit drinks	0.0	0.0	0.0	0.0	0.0	0.0	26.8	25.1	26.7	33.8	21.4	26.8	17	20	20	23	19	20
Sweet snack bars	1.8	1.7	1.4	1.9	1.2	1.6	5.3	5.6	5.4	6.2	4.4	5.7	35	41	41	45	44	42
Luncheon meats	0.9	1.7	1.9	2.8	2.5	2.8	0.1	0.2	0.3	0.4	0.3	0.3	278	437	505	763	677	530
Cordials	0.0	0.0	0.0	0.0	0.0	0.0	30.4	41.8	37.4	45.2	35.1	38.9	25	33	26	34	22	29
Butter and dairy fats	3.5	3.5	4.1	7.8	3.3	4.3	0.0	0.0	0.0	0.0	0.0	0.0	45	48	56	113	45	59
Lollies and confectionary	0.1	0.1	0.5	0.3	0.0	0.2	9.7	11.1	15.2	20.9	11.8	13.9	21	23	30	45	25	29
All sugar-sweetened drinks	0.0	0.0	0.0	0.0	0.0	0.0	29.5	33.5	42.0	56.0	56.2	44.2	25	37	52	75	83	56
All discretionary choices	8.0	12.3	16.6	19.3	15.6	14.6	26.3	40.7	57.3	68.7	66.7	51.4	512	841	1023	1267	1132	959
